# Physical Activity Dimensions and Its Association with Risk of Diabetes in Middle and Older Aged Chinese People

**DOI:** 10.3390/ijerph17217803

**Published:** 2020-10-25

**Authors:** Zixin Zeng, Yuqian Bian, Yiran Cui, Donghui Yang, Yafeng Wang, Chuanhua Yu

**Affiliations:** 1 Department of Epidemiology and Biostatistics, School of Health Sciences, Wuhan University, Wuhan 430071, China; zzx7021@whu.edu.cn (Z.Z.); 2019283050055@whu.edu.cn (Y.C.); yangdh@whu.edu.cn (D.Y.); wonyhfon@whu.edu.cn (Y.W.); 2 Information Management and Information System, School of Medical and Health Management, Huazhong University of Science and Technology, Wuhan 430071, China; byq847660458@163.com

**Keywords:** diabetes risk, physical activity, middle- and older-aged individuals

## Abstract

*Background:* Diabetes and physical inactivity are prevalent worldwide. Risk of diabetes is known to be related with insufficient physical activity (PA), but associations with the respective dimensions of PA is unclear. *Objective:* To describe the patterns of physical activity among Chinese middle- and older-aged individuals and figure out their associations with diabetes risk in different dimensions. *Methods:* Extracting self-reported data from China Health and Retirement Longitudinal Study (CHARLS, 2015), this study included 6196 participants. Multivariate logistic regression was conducted to determine the association between diabetes risk and PA dimensions such as intensity, frequency, duration, and volume. *Results:* Concerning frequency, lower diabetes risk was associated with performing vigorous PA at any frequency overall. For duration, smaller odds of diabetes were observed in performing vigorous PA 2–4 h/day (OR 0.46, 95%CI 0.30 to 0.71), moderate PA ≥4 h/day (OR 0.59, 95%CI 0.42 to 0.82) and light PA ≥4 h/day (OR 0.59, 95%CI 0.41 to 0.85) overall. For volume, lower diabetes risk was associated with performing moderate-to-vigorous PA (MVPA) ≥2250 METs/week (OR 0.58, 95%CI 0.42 to 0.81) in middle-aged group (45–64 years), whereas no significant associations between MVPA and diabetes risk were found in older aged group (≥65 years). *Conclusions:* Our results revealed that physical inactivity is prevalent in China, with a greater proportion in the diabetes group. Lower risk of diabetes was associated with higher frequency, longer duration and longer volume of PA at higher intensity in middle-aged respondents and similar associations at lower intensity for the older adults. Additionally, further well-designed prospective studies are needed to confirm our findings.

## 1. Introduction

Diabetes is one of the leading causes of death worldwide, resulting in an estimated 4.2 million deaths in 2019 [1]. Globally, it is estimated there wil be 578 million diagnosed diabetes cases in 2030, rising to 700 million by 2045 [2]. Due to the changing lifestyles and an aging population, China has become a major area of the rapidly emerging epidemic of diabetes with the largest number of older residents with diabetes [3,4]. Policies to control population levels of diabetes risk are projected to be prioritized urgently.

It is well established that physical activity (PA) is closely related to fitness and beneficial to lower the risks of type 2 diabetes mellitus (T2DM) and other chronic diseases [5]. Type 2 diabetes accounts for 90% of diabetes cases and is largely the result of excess body weight and physical inactivity [6]. Regular physical activity, including aerobic activity and muscle-strengthening activity, is essential for healthy aging. A recent systematic review of 53 studies that evaluated 66 lifestyle intervention programs reported that diet and physical activity promotion programs reduced type 2 diabetes incidence and other health-related outcomes compared with usual care [7]. Nonetheless, a population-based study [8] declared that more than a quarter of global adults were not getting enough physical activity in 2016. Although the prevalence of leisure-time physical activity (LTPA) and the proportion of residents to reach the vigorous-intensity level had been increasing among adults aged 18 years and above, they were still at a low level according to China Health and Nutrition Survey. [9] Limited by comorbidity and aging factors, a greater number of middle and older aged adults failed to achieve the goal of keeping physically active.

Physical activity mainly includes four dimensions: intensity, frequency, duration and mode. The American Heart Association has defined intensity as rate of energy expenditure which is an indicator of the metabolic demand of PA. Frequency is defined as number of sessions per day or per week and is usually qualified as number of bouts ≥10 min in duration. Duration is interpreted as time (minutes or hours) of PA bout during a specified time scale (e.g., day, week, year or past month) [10]. In terms of domain, physical activity is composed of occupational, transportation, leisure-time and household physical activity [10]. A considerable amount of research has consistently manifested that higher levels of LTPA showed apparent protective effects on the risk of diabetes compared with inactive individuals [11], and extended LTPA reduced diabetes risk furthermore [12]. In contrast, the association between physical activity with other domains and diabetes risk remains unclear [13]. The 2018 Physical Activity Guidelines for Americans recommend that older individuals perform at least 150–300 min of moderate-intensity or 75–150 min of vigorous-intensity aerobic physical activity per week in account of any domain. Even though compelling evidence supports a causal relationship between physical inactivity and the increasing risk of diabetes, excessive total physical activity being harmful to diabetes risk is understudied among middle- and older-aged adults in China and the respective PA dimensions’ influence on the risk of diabetes awaits statistical validation.

On the basis of these results, this study aims to describe the status of physical activity among Chinese middle- and older-aged individuals and evaluate the association of PA and its various dimensions (intensity, frequency, duration and volume) with the risk of diabetes.

## 2. Materials and Methods

### 2.1. Study Population

The China Health and Retirement Longitudinal Study (CHARLS) is a longitudinal study assessing the health, social and economic status of nationally representative samples which cover 450 villages and 150 counties in 28 provinces. The baseline survey was performed in 2011–2012 and follow-up surveys were conducted every other year. The survey objects are Chinese community-dwelling adults aged 45 or older and their spouse. A detailed description of the survey design and methods has been published previously [14]. This current cross-sectional study adopts the most recent survey (Wave 4) data available. The Biomedical Ethics Review Committee of Peking University approved CHARLS, and the ethical approval number was IRB00001052-11015. All participants were requested to provide written informed consent.

To be included in this study, participants (age ≥45 years) should have complete data on gender, educational level, marital status, smoking status, drinking status, body mass index (BMI), physical activity record, diabetes record and case weight. Then we excluded the samples with abnormal value (BMI > 100) or logic errors. Finally, 6196 participants were included in the current study.

### 2.2. Outcome Variable

The definition of diabetes is as follows: self-reported diabetes mellitus diagnosed by doctors (Questionnaire item: “Have you been diagnosed with diabetes or high blood sugar?”, and further information on diabetes medication history), fasting glucose ≥126 mg /dL or glycosylated hemoglobin ≥6.5% found in a blood test [15], and meeting any of the criteria is deemed as having diabetes.

### 2.3. Assessment of Physical Activity

Respondents completed a questionnaire where they reported weekly PA in three predefined categories followed by quintessential examples: (1) vigorous physical activity (VPA): activities that force you to breathe much faster or deeper than usual and may include heavy lifting, fast bicycling, digging or aerobics; (2) moderate physical activity (MPA): activities that compel you to breathe somehow harder than normal and may include carrying light loads, mopping the floor, Tai Chi or brisk walk; (3) light physical activity (LPA): activities that includes walking at home and at work. The questionnaire started with “did you do any VPA for at least 10 min continuously?”, more details would be asked if the answer is yes while skip to PA of lower intensity if the answer for no.

Then two more questions were raised to assess physical activity in self-reported questionnaire. First, participants were asked “During a usual week, on how many days did you do VPA/MPA/LPA for at least 10 min?” with the answer ranged from 1 to 7. The frequency was calculated by multiplying the days conducting VPA/MPA/LPA at least 10 min in a usual week and categorized into four groups (no activity, 1–2 days per week, 3–5 days per week, 6–7 days per week). Second, participants were asked “How much time did you usually spend doing VPA/MPA/LPA on one of those days?” So the duration of VPA/MPA/LPA was defined as the length of PA conducted in one day, and classified in accordance with CHARLS by five levels (no activity, 10–29 min per time, 30–119 min per time, 120–239 min per time, ≥240 min per time). Last, the volume was generated by frequency and duration (i.e., volume = frequency × duration). Given that the questionnaire didn’t refer to concrete duration of each time, average value was applied to produce the volume of VPA/MPA/LPA instead. According to the 2018Physical Activity Guidelines for Americans, the volume of VPA was divided into four scales (no activity, 10–74 min per week, 75–299 min per week, ≥300 min per week) and the volume of MPA was divided into four levels (no activity, 10–149 min per week, 150–299 min per week, ≥300 min per week). Regrettably, we used quartile as cut-offs in view of no authoritative volume classification for LPA (no activity, 10–105 min per week, 106–525 min per week, 526–1260 min per week, >1260 min per week).

Metabolic equivalent of task (MET) was cited to calculate the volume of moderate-to-vigorous-intensity physical activity (MVPA) with considerations of intensity. One MET represents the resting energy expenditure during quiet sitting. In our study, one minute of VPA is equivalent to 7.5 METs, and 4.5 METs for MPA [9]. The volume of MVPA was classified by the minimum and maximum of the recommendation in the guideline (no activity, 45–675 METs, 676–2250 METs, >2250 METs).

### 2.4. Covariates

A set of covariates were included: (1) demographic variables: gender (1. male, 2. female), age, educational level (1. junior high school or below, 2. senior high school and vocational school, 3. college or higher), marital status (1. married or partnered, 2. separated, divorced or widowed, 3. never married) (2) health behaviors: drinking (1. never drinks, 2. quit drinking 3. still drinks), smoking (1. never smokes, 2. quit smoking, 3. still smokes) (3) health-status related variables: BMI (1. underweight, 2. normal, 3. overweight, 4. obese). Age was treated as continuous variables, while gender, marital status, education, drinking, smoking and BMI were treated as categorical variables.

### 2.5. Data Analysis

Frequency and case weighted percentage were calculated to describe sociodemographic parameters and level distributions of physical activity among participants. A logistic regression analysis was used to determine the associations between various dimension of PA and diabetes. Key outcomes were presented by Odds Ratio and 95% Confidence Interval (OR and 95% CI) adjusted for seven potential confounders. In sensitivity analysis, we conducted the same analysis for the whole sample excluding participants with disability (2.80%) to reduce influences of restricted movement. Additional E-value was calculated to measure an association’s robustness to potential uncontrolled confounders. A larger E-value implies that considerable unmeasured confounding would be needed to explain away an effect estimate [16]. All statistical analyses were performed in Stata version 15.0 (Stata Corp, College Station, TX, USA). Case weights were offered by CHARLS and statistical significance was set at *p* < 0.05 (two-tailed) for all analyses.

## 3. Results

### 3.1. Demographic Characteristics

Table 1 presented the parameters for demographics, health behaviors and health-related variable. Application of the inclusion and exclusion criteria resulted in 6196 respondents from CHARLS (2015) being enrolled in this study, of which greater proportion were female, in middle-aged group, with 9th grade education or less, married or living with a partner, non-smokers, non-drinkers, in normal weight and involved in physical activities. Particularly, 687 diabetes cases (11.54%) and 5509 non-diabetes cases (88.46%) yield similar results.

### 3.2. Volume of PA

**Figure 1:**
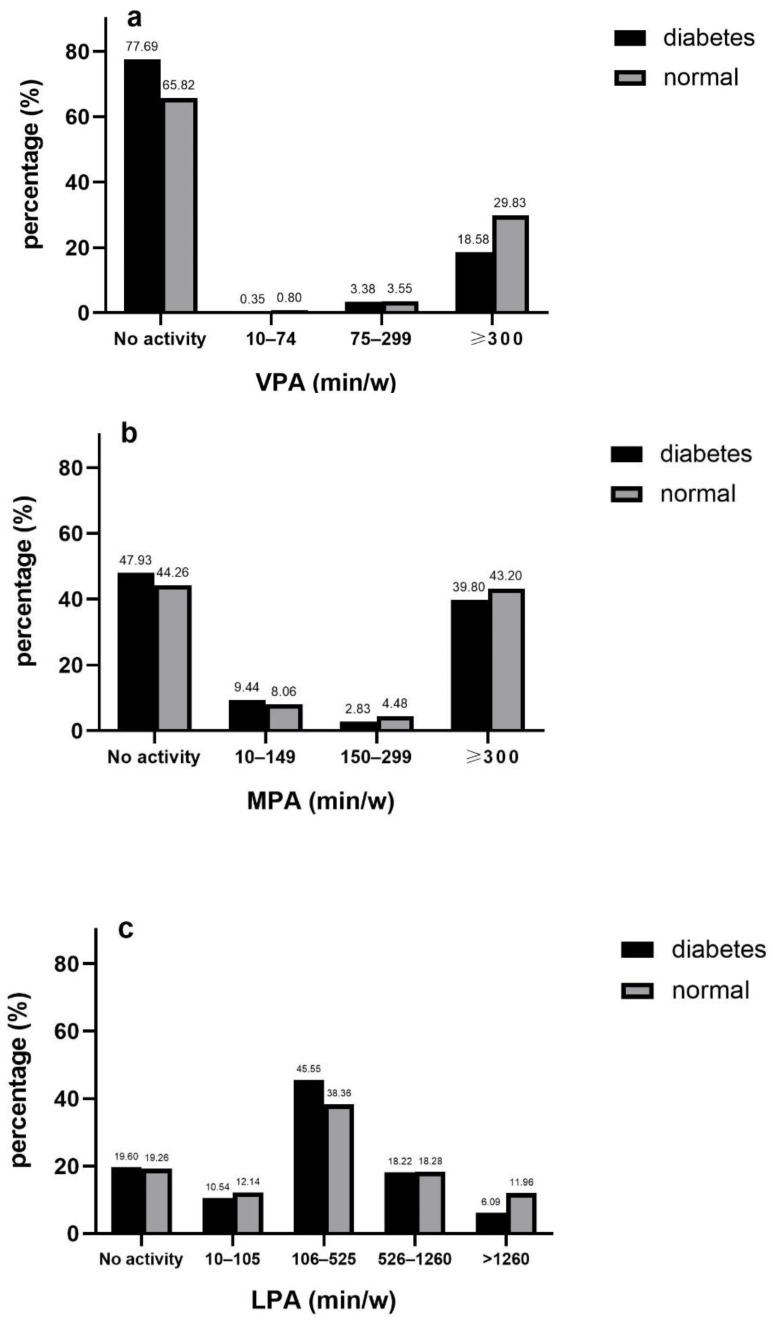

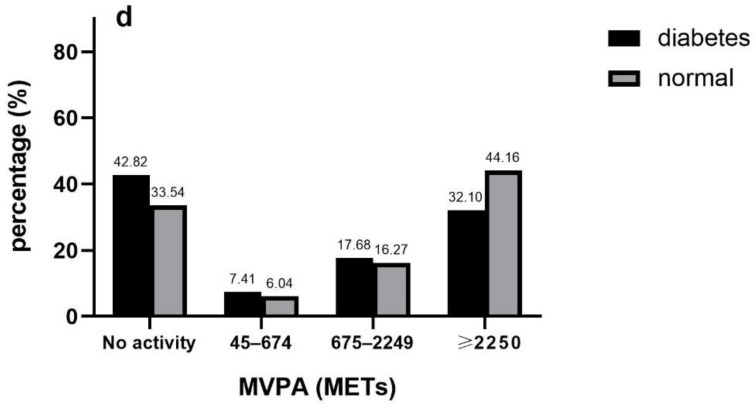
Volume of VPA (**a**), MPA (**b**), LPA (**c**) and MVPA (**d**) in diabetic and non-diabetic participants.

The distributions of VPA and MPA were heavily bipolarized, which a larger proportion of respondents was inactive or belonged to highest level. The proportion of each level was relatively evenly distributed with regard to LPA and MVPA. People were predominantly engaged in physical activity for 105–525 min per week at low intensity (39.19%), and individuals not being enrolled in MVPA took up the greatest proportion (Figure 1a–d).

The prevalence of diabetes was 36% lower in participants with a physical activity level of over 300 min/week for VPA overall (OR 0.64, 95%CI 0.50 to 0.83) and 47% lower in middle age (OR 0.53, 95%CI 0.39 to 0.72). Similarly, the prevalence of diabetes was 44% lower in subjects with a level over 1260 min/week in reference to MPA (OR 0.56, 95%CI 0.34 to 0.91) and 56% lower in middle age (OR 0.44, 95%CI 0.22 to 0.87). 

### 3.3. Frequency of PA

**Figure 2:**
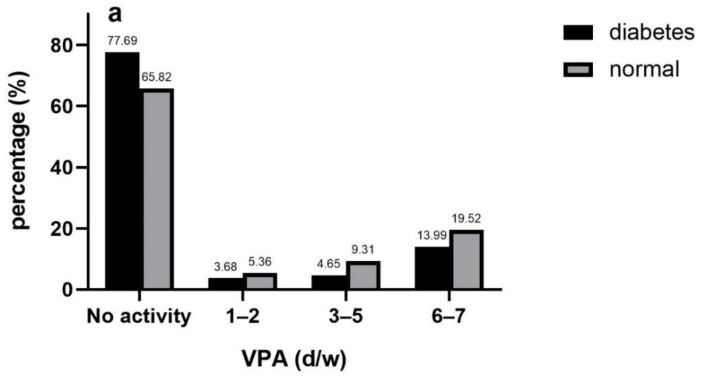

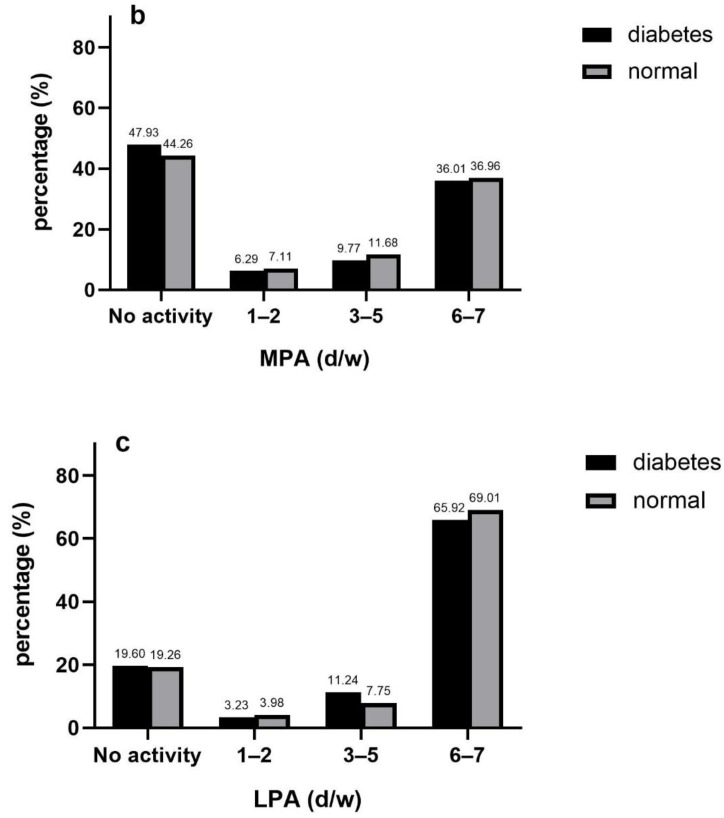
Frequency of VPA (**a**), MPA (**b**) and LPA (**c**) in diabetic and non-diabetic participants.

In the overwhelming majority of participants had no activities or conducted frequent physical activities (6–7 days/week) in all intensities, and two subgroups were both more likely to choose PA of lower intensity. Proportion distributions in diabetes group and non-diabetes group were similar in any single intensity. In diabetes group, respondents conducted VPA, MPA and LPA accounted for 22.31%, 52.07% and 80.4% while the percentage of conducting VPA, MPA and LPA in the normal group was 34.18%, 55.74% and 80.74%, respectively (Figure 2a–c).

Compared with inactive respondents, the risks of diabetes in the whole sample who conducted VPA 1–2 days/week, 3–5 days/week and 6–7 days/week were reduced by 36% (OR 0.64, 95%CI 0.41 to 0.99), 50% (OR 0.50, 95%CI 0.32 to 0.79) and 24% (OR 0.76, 95%CI 0.58 to 0.99), respectively. No significant relations between frequency of MPA/LPA and diabetes were observed. Similar patterns were discovered in middle-aged group but only conducting VPA 3–5days/week was associated with risk reduction of diabetes in old-aged group (Table 2).

### 3.4. Duration of PA

**Figure 3:**
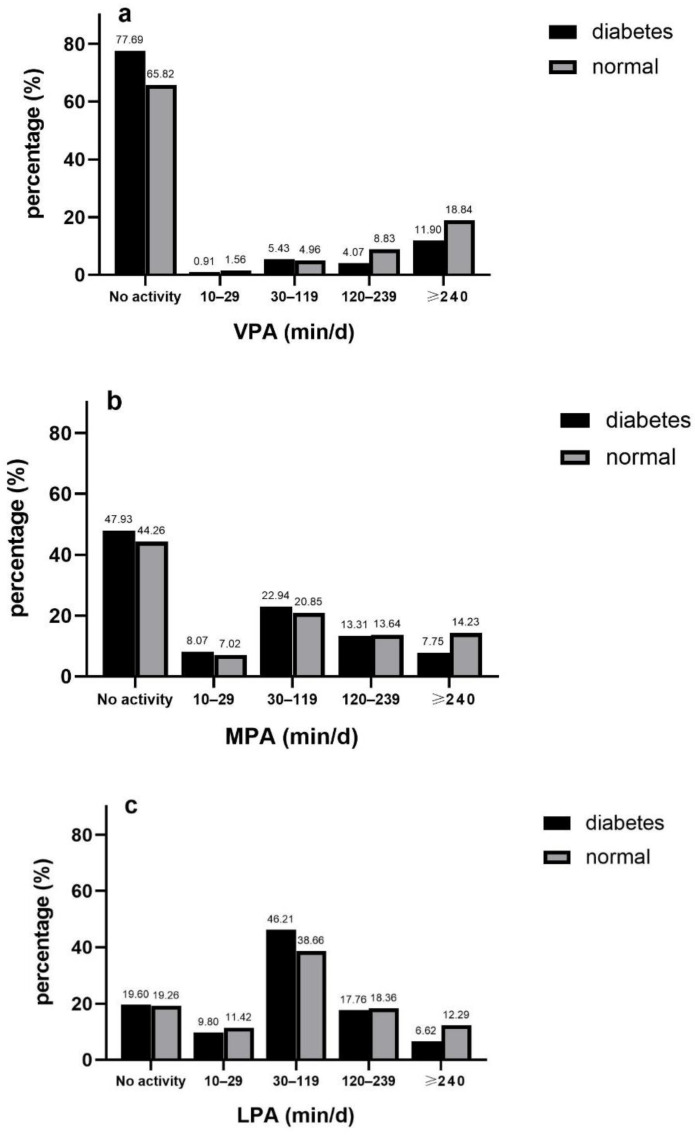
Duration of VPA (**a**), MPA (**b**) and LPA (**c**) in diabetic and non-diabetic participants.

Most of the participants didn’t conduct VPA or MPA (67.19% for VPA, 44.68% for MPA), but they preferred performing LPA for 30–119 min per day (39.53%). Among subjects active in VPA, proportion increased with the length of duration raised except a marginal rebound in the level of “120–239 min/day” in diabetes group. Regarding MPA and LPA, participants were more likely to perform physical activities during 30–119 min/day. Distribution discrepancies were also observed in MPA and LPA between two groups. The active respondents with diabetes conducting physical activities over 240 min per day took the smallest account (7.75% for MPA, 6.62 for LPA) but that less than 30 min per day took the smallest account (7.02 for MPA, 11.42 for LPA) in normal respondents (Figure 3 a–c).

Lower risk of diabetes was observed in respondents spending 2–4 h (OR 0.46, 95%CI 0.30 to 0.71) or over 4 h (OR 0.67, 95%CI 0.51 to 0.89) for VPA. Evident protecting effects were showed in middle-aged group but not in old-aged group. Spending over 4 h on MPA each time was correlated with smaller odds of diabetes (OR 0.59, 95%CI 0.42 to 0.82) and two age-stratified subgroups were in consistence. Participants performing 4 h LPA each time had lower risk of diabetes (OR 0.59, 95%CI 0.41 to 0.85). Reverse associations with long duration of diabetes were strong in elder-aged group while it was not statistically significant in the other subgroup (Table 2).

### 3.5. Sensitivity and Subgroup Analysis

Appendix ATable A1 presents similar results when respondents with disability were excluded. Frequency of VPA and diabetes risk had ORs of at least 2.50, 3.41 and 1.96 beyond the measured confounders, which indicated that the observed OR of 0.64, 0.50 and 0.76 could be explained by an unmeasured confounder that was correlated with both frequency-level vigorous physical activity and lower diabetes risk by an OR of 2.50, 3.41 and 1.96 each, over and above the measured confounder. More E-value results for the point estimate were listed in Appendix A Table A2. In the gender-stratified analyses, a similar inverse relationship was observed in females and the results in males are less significant in Appendix A Table A3.

## 4. Discussion

In total, the majority of participants with or without diabetes do not reach the recommended frequency or length of physical activity. More frequent PA (>0 day/week) are associated with lower diabetes risk at vigorous intensity only, while inverse associations between duration and volume and diabetes risk are significant in all intensities. Lower diabetes risk seems to be associated with long-duration and high-volume of VPA/MPA in middle-aged individuals (45–64 years), and long-duration and high-volume of MPA/LPA for the older adults (≥65 years).

A cross-sectional survey by He et al. [17] indicates that nearly 50% of diabetes subjects didn’t meet minimum PA recommendations, whereas the number raised to almost 60% in our study. He’s survey was carried out in 12 hospitals and targeted outpatient populations with higher attention to lifestyle interventions compared to community-dwelling participants, which may partly explain the numerical discrepancy. The guidelines [5] recommend that all older adults including those with chronic diseases perform activities on at least 3 days a week to produce substantial health benefits and 150 cumulative minutes of moderate aerobic PA, or at least 75 min of vigorous aerobic PA, or an equivalent combination of moderate- and vigorous-intensity aerobic activity per week are essential as well. In the present study, most of participants did not reach the recommended frequency or volume of PA with or without diabetes. A cross-sectional study [18] conducted in Shenzhen, a city in southern China, affirmed that 63.1% community residents aged more than 40 years were physical inactive with a higher proportion in the older age group. A systematic review [19] targeted to older adults showed certain pivotal barriers for PA. On the one hand, internal factors may account for low-level of PA, including physical limitations (e.g., pain or discomfort and lack of strength, balance or flexibility), negative perception (e.g., apathy and fear of injury) and existing sedentary habits. On the other hand, external factors may also influence physical activity behaviors, including lack of social support (e.g., little interaction with peers, social awkwardness and lack of encouragement), access difficulties (e.g., extreme environments and high costs) and absence of professional instruction. Due to the reasons mentioned above, the majority of middle and older aged adults are performing insufficient physical activity without plans or monitors [20,21].

In the current study, both the descriptive and regression analyses revealed that the higher engagement in all dimensions indicated a positive relationship with diabetes risk. All older adults experience a loss of physical functional capacity, while some are in better condition with lifelong physically activity [22]. It is biologically plausible that physical activity is correlated with lower diabetes risk since it reduces insulin-mediated and non–insulin-mediated glucose disposal [23,24]. In the present study, participants who are involved in VPA at least one day a week have smaller odds ratio of diabetes risk. A recent population-based survey [25] showed that participants who perform physical activity at any frequency had significant lower risk of all-cause mortality. The guideline stress particular emphasize on benefits of conducting physical activity 3–7 days per week for older individuals. Another recent research [26] also reports that frequency of ≥3 times in weekly leisure-time running is correlated with lower diabetes risk, which is similar to our results. As for duration, our results indicate that being involved in at least 120-min VPA, 240-min MPA or 240-min LPA each day is associated with attenuated risk of diabetes for the whole sample. Among the limited number of studies without conversion to volume, a randomized controlled trial (RCT) demonstrates that at least 3 h per day for LPA improved glucose control in older adults [27]. Furthermore, another RCT [28] conducted among diabetic individuals reveals that HbA1c decreased ~1% on average when respondents were engaged in additional LPA >55.2 min/day or MVPA >7.33 min/day than normal. Despite the different definitions and cutoff points of duration, higher level of duration is inversely associated with diabetes risk.

Regarding volume, a meta-analysis determines that achieving recommended volume of PA is associated with a 26% reduction in risks for diabetes mellitus incidence [29]. In this study, performing over 2250 METs of MVPA (equivalent to 300 min of VPA) weekly has protective associations with diabetes risk. Recent studies show that increasing length of MVPA is associated with a reduction in diabetes mellitus risk indicators such as HbA1c, particularly in subjects with dysglycemia [30]. In addition, our study finds that sufficient volume of MPA (150–300 min per week according to recommendations) shows significantly positive associations with the risk of diabetes, but not for sufficient VPA. Inconsistent with our results, a cohort study [31] controlling the dose of physical activity suggests that VPA alone or VPA combined with MPA yield stronger health profits in terms of diabetes risk reduction compared with MPA alone. Instead of total physical activity, the cohort study includes only the effects of leisure-time exercise on diabetes risk, which may attributes to the differential effects of VPA and MPA [13]. However, engaging in vigorous physical activity up to 300 min a week attained profits in the present study, which shows higher threshold for physical activity profits compared with recommendations. In our study, lower risk of diabetes is associated with higher PA level even at low intensity, which is consistent with previous studies [32]. Subgroup differences exist in every PA dimension, PA tends to be related with diabetes risk at lower intensity in the older aged group. A National Health and Nutrition Examination Survey study [33] assessing the effects of leisure-time physical activity on undetected prediabetes, suggests that high level of physical activity at higher intensity have protective effects on prediabetes among 45 to 65 age group, which yield similar results with ours.

The strengths of this study include the use of a nationwide representative sample covering 28 provinces in mainland China and the CHARLS database has been adopted in a large number of high-quality studies to prove its validity and reliability [14]. This study also has certain limitations. First, cross-sectional study can’t explain the causal association between PA and diabetes. Second, recall bias is avoidable in self-reported questionnaires. A Canadian survey stated that self-reported physical activity is overestimated on average compared to the volume accumulated on an accelerometer [34], therefore objective measurements are needed beyond self-reported questionnaires for more accurate physical activity data [35]. Third, LPA only includes walking in the current study while more PA items under 3 MET should be added to ensure variable accuracy [36,37].

## 5. Conclusions

Our results revealed that both diabetic and non-diabetic individuals were performing lower level of physical activity than recommendation, and participants with diabetes were conducting more insufficient PA. Concerning frequency, only VPA is associated with lower diabetes risk. For duration and volume, the inverse associations between diabetes and total PA were strong in middle-aged group in VPA/MPA, and that were significant in MPA/LPA for the older-aged group. The findings from this study extend previous cross-sectional evidence and further well-designed prospective studies using a more accurate assessment of physical activity such as the accelerometer are warranted to evaluate the association of physical activity dimensions with risk of diabetes.

## Figures and Tables

**Table 1:** Participants characteristics stratified by diabetes.

Variables	Total (*n* = 6196)	Diabetes (*n* = 687)	Non-Diabetes (*n* = 5509)
Gender			
female	3290(52.62)	404(58.62)	2886(51.84)
male	2906(47.38)	283(41.38)	2623(48.16)
Age			
45–64	4121(66.06)	430(61.70)	3691(66.63)
65–93	2075(33.94)	257(38.30)	1818(33.37)
Educational level			
junior high school or less	4231(65.61)	464(64.27)	3767(65.78)
senior high school and vocational school	1870(32.06)	206(31.42)	1664(32.14)
college or higher	95(2.34)	17(4.30)	78(2.08)
Marital status			
married or cohabiting	5427(87.19)	597(86.23)	4830(87.31)
separated, divorced or widowed	741(12.29)	88(13.53)	653(12.13)
never married	28(0.52)	2(0.24)	26(0.56)
Drinking			
never drinks	3375(53.33)	390(54.43)	3070(53.19)
quit drinking	687(10.99)	106(17.10)	837(10.20)
still drinks	2134(35.67)	191(28.47)	1602(36.61)
Smoking			
never smokes	3483(55.98)	413(59.02)	2985(55.58)
quit smoking	981(16.36)	144(20.49)	581(15.82)
still smokes	1732(27.66)	130(20.49)	1943(28.60)
BMI			
underweight	369(5.88)	24(3.58)	345(6.18)
normal	3594(57.80)	308(45.51)	3286(59.41)
overweight	1896(31.28)	288(42.63)	1608(29.79)
obese	337(5.04)	67(8.27)	270(4.62)
PA-performing			
no	669(9.91)	96(13.12)	573(9.49)
yes	5527(90.09)	591(86.88)	4936(90.51)

**Table 2:** Associations between diabetes risk and PA frequency, duration and volume.

	Model 1		Model 2 Middle Age		Model 3 Old Age	
	OR	95%CI	OR	95%CI	OR	95%CI
**Frequency**						
**VPA**						
No activity	0.00		0.00		0.00	
1–2 d/w	0.64 *	0.41, 0.99	0.54 *	0.32, 0.93	0.91	0.43, 1.93
3–5 d/w	0.50 **	0.32, 0.79	0.53 *	0.31, 0.92	0.35 **	0.16, 0.77
6–7 d/w	0.76 *	0.58, 0.99	0.60 **	0.43, 0.84	1.16	0.75, 1.81
**MPA**						
No activity	0.00		0.00		0.00	
1–2 d/w	0.88	0.61, 1.26	0.76	0.47, 1.22	1.13	0.62, 2.06
3–5 d/w	0.82	0.60, 1.12	0.74	0.50, 1.11	0.94	0.57, 1.54
6–7 d/w	0.94	0.73, 1.20	0.94	0.68, 1.30	0.91	0.64, 1.29
**LPA**						
No activity	0.00		0.00		0.00	
1–2 d/w	0.78	0.49, 1.26	1.00	0.57, 1.77	0.53	0.23, 1.22
3–5 d/w	1.38	0.85, 2.22	1.61	0.86, 3.03	0.83	0.55, 1.98
6–7 d/w	0.93	0.73, 1.17	0.98	0.73, 1.31	0.83	0.57, 1.22
**Duration**						
**VPA**						
No activity	0.00		0.00		0.00	
10–29 min/d	0.59	0.26, 1.34	0.48	0.19, 1.24	1.30	0.29, 5.89
30–119 min/d	0.97	0.62, 1.53	1.10	0.65, 1.88	0.74	0.31, 1.78
120–239 min/d	0.46 ***	0.30, 0.71	0.42 **	0.24, 0.71	0.57	0.29, 1.12
≥240 min/d	0.67 **	0.51, 0.89	0.52 ***	0.37, 0.73	1.14	0.73, 1.80
**MPA**						
No activity	0.00		0.00		0.00	
10–29 min/d	1.10	0.78, 1.55	1.05	0.68, 1.63	1.25	0.71, 2.21
30–119 min/d	0.99	0.72, 1.36	0.99	0.64, 1.53	0.96	0.64, 1.44
120–239 min/d	0.98	0.73, 1.32	0.92	0.64, 1.34	1.06	0.66, 1.69
≥240 min/d	0.59 **	0.42, 0.82	0.58 **	0.39, 0.87	0.56	0.31, 1.00
**LPA**						
No activity	0.00		0.00		0.00	
10–29 min/d	0.82	0.59, 1.14	0.93	0.61, 1.40	0.70	0.40, 1.23
30–119 min/d	1.12	0.86, 1.47	1.29	0.91, 1.84	0.87	0.58, 1.32
120–239 min/d	0.94	0.70, 1.27	0.80	0.54, 1.18	1.13	0.71, 1.80
≥240 min/d	0.59 **	0.41, 0.85	0.75	0.49, 1.14	0.33 **	0.15, 0.69
**Volume**						
**VPA**						
No activity	0.00		0.00		0.00	
10–74 min/w	0.44	0.13, 1.46	0.58	0.17, 1.93	N/A	
75–299 min/w	0.88	0.49, 1.57	0.90	0.46, 1.79	0.74	0.25, 2.24
≥300 min/w	0.64 **	0.50, 0.83	0.53 ***	0.3, 0.72	0.92	0.63, 1.35
**MPA**						
No activity	0.00		0.00		0.00	
10–149 min/w	1.11	0.80, 1.54	1.11	0.73, 1.67	1.11	0.64, 1.93
150–299 min/w	0.56 *	0.34, 0.91	0.44 *	0.22, 0.87	0.74	0.31, 1.73
≥300 min/w	0.91	0.71, 1.15	0.88	0.64, 1.21	0.93	0.67, 1.29
**LPA**						
No activity	0.00		0.00		0.00	
10–105 min/w	0.83	0.60, 1.14	0.97	0.65, 1.45	0.66	0.38, 1.15
106–525 min/w	1.12	0.85, 1.47	1.27	0.89, 1.81	0.89	0.59, 1.35
526–1260 min/w	0.97	0.73, 1.30	0.84	0.57, 1.24	1.12	0.71, 1.79
>1260 min/w	0.56 **	0.38, 0.81	0.70	0.46, 1.09	0.32 **	0.15, 0.69
**MVPA**						
No activity	0.00		0.00		0.00	
45–675 METs	0.99	0.69, 1.42	0.95	0.60, 1.51	1.01	0.54, 1.87
676–2250 METs	0.85	0.58, 1.24	0.78	0.47, 1.31	0.98	0.63, 1.52
≥2250 METs	0.67 **	0.52, 0.87	0.58 **	0.42, 0.81	0.82	0.56, 1.18

Note: ORs were adjusted for age, gender, marital status, education, drinking, smoking and BMI level. N/A means no applicable value is observed in the calculation. * *p* < 0.05; ** *p* < 0.01; *** *p* < 0.001.

## Appendix A

**Table A1:** Associations between diabetes risk and PA frequency, duration and volume (exluding disability).

	Model1	
	OR	95%CI
**Frequency**		
**VPA**		
No activity	0.00	
1–2 d/w	0.68	0.44, 1.07
3–5 d/w	0.51 **	0.31, 0.84
6–7 d/w	0.73 *	0.54, 0.98
**MPA**		
No activity	0.00	
1–2 d/w	0.86	0.58, 1.26
3–5 d/w	0.85	0.61, 1.19
6–7 d/w	0.97	0.74, 1.26
**LPA**		
No activity	0.00	
1–2 d/w	0.82	0.50, 1.34
3–5 d/w	1.45	0.88, 2.39
6–7 d/w	0.90	0.70, 1.15
**Duration**		
**VPA**		
No activity	0.00	
10–29 min/d	0.67	0.30, 1.53
30–119 min/d	1.06	0.66, 1.71
120–239 min/d	0.44 **	0.28, 0.71
≥240 min/d	0.64 **	0.48, 0.87
**MPA**		
No activity	0.00	
10–29 min/d	1.06	0.73, 1.54
30–119 min/d	1.02	0.73, 1.43
120–239 min/d	1.02	0.74, 1.41
≥240 min/d	0.61 **	0.43, 0.86
**LPA**		
No activity	0.00	
10–29 min/d	0.84	0.59, 1.18
30–119 min/d	1.12	0.84, 1.49
120–239 min/d	0.91	0.66, 1.24
≥240 min/d	0.55 **	0.37, 0.82
**Volume**		
**VPA**		
No activity	0.00	
10–74 min/w	0.48	0.14, 1.63
75–299 min/w	0.91	0.50, 1.66
≥300 min/w	0.59 ***	0.46, 0.75
**MPA**		
No activity	0.00	
10–149 min/w	0.97	0.69, 1.38
150–299 min/w	0.57 *	0.35, 0.94
≥300 min/w	0.88	0.69, 1.14
**LPA**		
No activity	0.00	
10–105 min/w	0.86	0.62, 1.21
106–525 min/w	1.12	0.84, 1.49
526–1260 min/w	0.94	0.69, 1.29
>1260 min/w	0.50 **	0.33, 0.75
**MVPA**		
No activity	0.00	
45–675 METs	0.85	0.58, 1.26
676–2250 METs	0.84	0.57, 1.24
≥2250 METs	0.61 ***	0.48, 0.79

Note: ORs were adjusted for age, gender, marital status, education, drinking, smoking and BMI level. * *p* < 0.05; **: *p* < 0.01; ***: *p* < 0.001.

**Table A2:** The E-value for the point estimate.

	Model 1		e-Value	
	OR	95%CI	Point Estimate	CI
**Frequency**				
**VPA**				
No activity	0.00			
1–2 d/w	0.64 *	0.41, 0.99	2.500	1.111
3–5 d/w	0.50 **	0.32, 0.79	3.414	1.846
6–7 d/w	0.76 *	0.58, 0.99	1.960	1.111
**MPA**				
No activity	0.00			
1–2 d/w	0.88	0.61, 1.26	——	——
3–5 d/w	0.82	0.60, 1.12	——	——
6–7 d/w	0.94	0.73, 1.20	——	——
**LPA**				
No activity	0.00			
1–2 d/w	0.78	0.49, 1.26	——	——
3–5 d/w	1.38	0.85, 2.22	——	——
6–7 d/w	0.93	0.73,1.17	——	——
**Duration**				
**VPA**				
No activity	0.00			
10–29 min/d	0.59	0.26, 1.34	——	——
30–119 min/d	0.97	0.62, 1.53	——	——
120–239 min/d	0.46 ***	0.30, 0.71	3.771	2.167
≥240 min/d	0.67 **	0.51, 0.89	2.350	1.496
**MPA**				
No activity	0.00			
10–29 min/d	1.10	0.78, 1.55	——	——
30–119 min/d	0.99	0.72, 1.36	——	——
120–239 min/d	0.98	0.73, 1.32	——	——
≥240 min/d	0.59 **	0.42, 0.82	2.780	1.737
**LPA**				
No activity	0.00	0.59, 1.14		
10–29 min/d	0.82	——	——
30–119 min/d	1.12	0.86, 1.47	——	——
120–239 min/d	0.94	0.70, 1.27	——	——
≥240 min/d	0.59 **	0.41, 0.85	2.780	1.632
**Volume**				
**VPA**				
No activity	0.00			
10–74 min/w	0.44	0.13, 1.46	——	——
75–299 min/w	0.88	0.49, 1.57	——	——
≥300 min/w	0.64 **	0.50, 0.83	2.500	1.702
**MPA**				
No activity	0.00				
10–149 min/w	1.11	0.80, 1.54	——	——
150–299 min/w	0.56 *	0.34, 0.91	2.970	1.429
≥300 min/w	0.91	0.71, 1.15	——	——
**LPA**				
No activity	0.00			
10–105 min/w	0.83	0.60, 1.14	——	——
106–525 min/w	1.12	0.85, 1.47	——	——
526–1260 min/w	0.97	0.73, 1.30	——	——
>1260 min/w	0.56 **	0.38, 0.81	2.970	1.429
**MVPA**				
No activity	0.00			
45–675 METs	0.99	0.69, 1.42	——	——
676–2250 METs	0.85	0.58, 1.24	——	——
≥2250 METs	0.67 **	0.52, 0.87	2.350	1.564

Note: ORs were adjusted for age, gender, marital status, education, drinking, smoking and BMI level. *: *p* < 0.05.; **: *p* < 0.01.; ***: *p* < 0.001.

**Table A3:** Associations between diabetes risk and PA frequency, duration and volume.

	Model 1: Male		Model 2: Female	
	OR	95%CI	OR	95%CI
**Frequency**				
**VPA**					
No activity	0.00		0.00	
1–2 d/w	0.67	0.36, 1.23	0.61	0.33, 1.14
3–5 d/w	0.40 *	0.17, 0.94	0.60 *	0.37, 0.99
6–7 d/w	0.91	0.61, 1.35	0.62 *	0.42, 0.90
**MPA**				
No activity	0.00		0.00	
1–2 d/w	0.88	0.47, 1.63	0.87	0.56, 1.36
3–5 d/w	0.94	0.58, 1.54	0.72	0.48, 1.08
6–7 d/w	1.13	0.77, 1.66	0.83	0.60, 1.13
**LPA**				
No activity	0.00		0.00	
1–2 d/w	0.63	0.28, 1.45	0.87	0.48, 1.58
3–5 d/w	1.88	0.92, 3.85	0.97	0.59, 1.59
6–7 d/w	0.84	0.59, 1.20	1.00	0.73, 1.35
**Duration**				
**VPA**				
No activity	0.00		0.00	
10–29 min/d	0.36	0.08, 1.64	0.85	0.32, 2.26
30–119 min/d	1.26	0.67, 2.39	0.75	0.41, 1.38
120–239 min/d	0.40 *	0.20, 0.78	0.51 *	0.30, 0.89
≥240 min/d	0.75	0.49, 1.15	0.60 *	0.41, 0.88
**MPA**				
No activity	0.00		0.00	
10–29 min/d	1.14	0.62, 2.08	1.06	0.70, 1.62
30–119 min/d	1.06	0.67, 1.68	0.93	0.62, 1.41
120–239 min/d	1.31	0.83, 2.07	0.78	0.53, 1.14
≥240 min/d	0.76	0.46, 1.24	0.46 ***	0.30, 0.73
**LPA**				
No activity	0.00		0.00	
10–29 min/d	0.91	0.55, 1.50	0.77	0.49, 1.19
30–119 min/d	1.15	0.76, 1.76	1.11	0.78, 1.58
120–239 min/d	0.73	0.47, 1.14	1.10	0.75, 1.62
≥240 min/d	0.52 *	0.29, 0.91	0.64	0.40, 1.03
**Volume**				
**VPA**				
No activity	0.00		0.00	
10–74 min/w	0.26	0.03, 2.10	0.60	0.13, 2.72
75–299 min/w	1.06	0.44, 2.55	0.74	0.37, 1.49
≥300 min/w	0.69	0.47, 1.01	0.60 **	0.43, 0.83
**MPA**				
No activity	0.00		0.00	
10–149 min/w	1.20	0.68, 2.14	1.04	0.70, 1.55
150–299 min/w	0.69	0.30, 1.57	0.47 *	0.25, 0.88
≥300 min/w	1.07	0.74, 1.55	0.80	0.59, 1.08
**LPA**				
No activity	0.00		0.00	
10–105 min/w	0.95	0.84, 1.55	0.76	0.49, 1.17
106–525 min/w	1.14	0.75, 1.75	1.11	0.78, 1.58
526–1260 min/w	0.73	0.47, 1.14	1.15	0.79, 1.68
>1260 min/w	0.51*	0.29, 0.91	0.59 *	0.36, 0.97
**MVPA**				
No activity	0.00		0.00		
45–675 METs	0.80	0.39, 1.61	1.06	0.69, 1.64
676–2250 METs	0.75	0.44, 1.28	0.90	0.56, 1.45
≥2250 METs	0.83	0.53, 1.30	0.56 ***	0.42, 0.75

Note: ORs were adjusted for age, gender, marital status, education, drinking, smoking and BMI level. *: *p* < 0.05; **: *p* < 0.01.; ***: *p* < 0.001.
